# Fowl Adenovirus (FAdV) Recombination with Intertypic Crossovers in Genomes of FAdV-D and FAdV-E, Displaying Hybrid Serological Phenotypes

**DOI:** 10.3390/v11121094

**Published:** 2019-11-26

**Authors:** Anna Schachner, Gabriel Gonzalez, Lukas Endler, Kimihito Ito, Michael Hess

**Affiliations:** 1 Christian Doppler Laboratory for Innovative Poultry Vaccines, University of Veterinary Medicine, 1210 Vienna, Austria; Michael.Hess@vetmeduni.ac.at; 2 Division of Bioinformatics, Research Center for Zoonosis Control, Hokkaido University, Sapporo 001-0020, Japan; gagonzalez@czc.hokudai.ac.jp (G.G.); itok@czc.hokudai.ac.jp (K.I.); 3 Bioinformatics and Biostatistics Platform, Department of Biomedical Sciences, University of Veterinary Medicine, 1210 Vienna, Austria; Lukas.Endler@vetmeduni.ac.at; 4 University Clinic for Poultry and Fish Medicine, Department for Farm Animals and Veterinary Public Health, University of Veterinary Medicine, 1210 Vienna, Austria

**Keywords:** fowl adenovirus, whole genome sequencing, recombination, hexon, fiber, cross-neutralization

## Abstract

After analyzing 27 new genomes from fowl adenovirus (FAdV) field isolates and so-far unsequenced prototypes, we report the first evidence for recombination in FAdVs. Recombination was confined to species FAdV-D and FAdV-E, accommodating the largest number of, and the intraspecies-wise most differentiated, types. The majority of detected events occurred in FAdV-E, involving segments with parental origin of all constitutive types. Together with the diversity of breakpoints, this suggests widespread recombination in this species. With possible constraints through species-specific genes and diversification patterns, the recombinogenic potential of FAdVs attains particular interest for inclusion body hepatitis (IBH), an important disease in chickens, caused by types from the recombination-prone species. Autonomously evolving, recombinant segments were associated with major sites under positive selection, among them the capsid protein hexon and fiber genes, the right-terminal ORFs 19, 25, and the ORF20/20A family. The observed mosaicism in genes indicated as targets of adaptive pressures points toward an immune evasion strategy. Intertypic hexon/fiber-recombinants demonstrated hybrid neutralization profiles, retrospectively explaining reported controversies on reference strains B3-A, T8-A, and X11-A. Furthermore, cross-neutralization supported sequence-based evidence for interdomain recombination in fiber and contributed to a tentatively new type. Overall, our findings challenge the purported uniformity of types responsible for IBH, urging more complete identification strategies for FAdVs. Finally, important consequences arise for *in vivo* studies investigating cross-protection against IBH.

## 1. Introduction

Fowl adenoviruses (FAdVs), belonging to the genus *Aviadenovirus* (family *Adenoviridae*), are mainly isolated from chickens, but sporadically also from other avian hosts [1]. The official classification recognizes five genomically distinct species, *Fowl aviadenovirus A* to *Fowl aviadenovirus E* (FAdV-A–FAdV-E), which are subdivided into types, determined by cross-neutralization (FAdV-1 to -8a, -8b to -11) and genotyping based on molecular criteria [1,2,3].

The advent of molecular FAdV typing was mainly endorsed by detection methods utilizing the major structural protein hexon, which could be widely harmonized with results from traditional serotyping [4,5]. As a fast tool for type inference, this revolutionized FAdV typing to the extent that hexon became the prevailing target for strain characterization in the last two decades.

Although the wealth of hexon data has critically contributed to the recognition of specific types for distinct clinical pictures [6,7], systematic intergenomic comparisons of FAdVs outside this approach are lagging far behind the state-of-art in mammalian, particularly human adenoviruses (HAdVs) of the genus *Mastadenovirus*.

Presently, eleven of the FAdV prototype genomes are completely sequenced (only a partial genome is available for FAdV-10, type member of species FAdV-C). In terms of diversification, FAdV-D and FAdV-E, which share close phylogenetic and biological relatedness (as etiological agents of the same disease, inclusion body hepatitis, i.e., IBH), represent exceptional species, accommodating the majority of unique FAdV serotypes and genotypes. However, the existing blueprint for type variety within FAdV-D and FAdV-E relies mostly on a single reference genome for each constitutive type [8], represented by apathogenic reference strains that were attenuated by long-term *in vitro* passaging since their initial isolation and characterization in the middle of the last century [9]. For genomes of contemporary field strains, there had so far only been singular contributions to public databases. Recently, this situation has changed for FAdV-4 (FAdV-C), with the sudden appearance of hepatitis-hydropericardium syndrome (HHS) in China, leading to an extensive supply of the relevant sequences [10,11,12].

Based on the known reference genomes for all species, and almost all types, it was observed that diversity in FAdVs is dictated by stereotypical variation in particular central genomic transcripts. Besides this, there are less well-characterized differences in nucleotide homology and ORF contents of the terminal regions [8,13], which comprise a substantial portion (ca. two thirds) of the total FAdV genome. Contents of the FAdV termini, particularly with regard to their transcription, expression, and functionality, are still poorly understood. However, with a mounting awareness that antigenic determinants alone cannot be used to reliably distinguish certain phenotypes, e.g., pathogenicity differences between strains of the same serotype, there is an increasing interest to elucidate terminal variation in FAdVs and how it relates to type specificity and virulence.

Recently we identified strains with contradictory type specificities in the most variable determinants, hexon loop-1 and fiber, which are not compatible with the existing scheme of genotypes [14]. This finding, for the first time, exposed shortcomings of methods for FAdV genotyping that rely exclusively on a single genomic region.

From a clinical viewpoint, this scrutinizes (i) the paradigm of specific diseases being linked to a restricted set of types, with types defined solely by hexon, and (ii) possible cross-protection between less well-characterized FAdVs in vaccination-challenge models.

Based on enlarged FAdV cohort genome data, we were able to determine a range of topological switches in the phylogenetic trees of informative genome regions, providing, for the first time, evidence for recombination and its systematic occurrence in FAdVs. The finding of recombination could be extended toward hitherto unsequenced reference strains, and a newly recruited field strain with interdomain exchange in its fiber. These results imply that circulating FAdVs, mainly from species FAdV-D and FAdV-E, are genetically more diverse than previously concluded from the available data. Additionally, we investigated consequences of antigenic reshuffling in recombinants by traditional cross-neutralization, considering for the first time all known reference strains of the examined FAdV species together in one setting.

## 2. Materials and Methods

### 2.1. Virus Strains and DNA Preparation

FAdV strains sequenced in this study were either isolated from documented clinical cases, or purchased from the American Type Culture Collection (Manassas, VA), as well as kindly supplied by H. M. Hafez (Institute of Poultry Diseases, Berlin, Germany) and Vaxxinova Japan K.K. (Tokyo, Japan) (Supplementary Table S1) [15,16,17,18,19,20,21,22,23,24,25,26,27,28,29]. Besides FAdV-D and FAdV-E strains, we sequenced further strains of species FAdV-A (including the hitherto unavailable OTE reference genome) and FAdV-C (including, for the first time, the complete FAdV-10 genome), in order to increase robustness of the analysis.

All viruses were 3-fold plaque purified and propagated in primary chicken-embryo liver (CEL) cells, as described by [30]. Cell culture supernatants were clarified by low-speed centrifugation (10 min, 2000× *g*), followed by virus concentration through ultracentrifugation; briefly, 5 mL of a 1.27 g/mL CsCl cushion were overlaid with 20 mL of clarified cell culture supernatant, and virions were pelleted through the cushion by ultracentrifugation (3 h, 141,000× *g*), using an SW 28 rotor (Beckman Coulter, Brea, CA, USA). The resulting pellets were used for DNA extraction with the DNeasy Blood & Tissue Kit (Qiagen, Hilden, Germany).

### 2.2. Sequencing and Genome Assembly

Whole-genome sequencing was performed with an Illumina system (MiSeq V3, Central Service Facility NGS Unit, Vienna, Austria). Paired-end libraries were generated, and virus samples were multiplexed in a single lane, separated by barcoding. After sorting out contaminating chicken genome reads by mapping the reads against the *Gallus gallus* genome (v. 5.0), the mitochondrial genome of *Gallus sonneratii* (AP006746.1), and against PhiX sequencing control (Illumina) to remove vector reads, only unmapped reads were used for the assembly of viral genomes [31]. Read sequences were trimmed to remove adapters and only reads >100 bp were considered in the assembly of the genomes.

For *de novo* assembly, reads were subsampled, and assembly was performed by using CLC Genomics Workbench v. 4.0 (CLC bio, Aarhus, Denmark). The resulting contigs were manually ordered and orientated by comparison with already available complete FAdV genomes. The contig sequences were aligned using Accelrys Gene version 2.5 (Accelrys, San Diego, CA).

Gaps between contigs and sequence ambiguities were resolved by Sanger sequencing (LGC Genomics, Berlin, Germany), with PCR primers designed on the flanking regions of sequences. Genome annotations were performed on the basis of already published genomes, using the NCBI ORF Finder (www.ncbi.nlm.nih.gov/gorf/orfig.cgi).

The complete genome sequences were deposited in GenBank, under accession numbers MK572847 to MK572875.

### 2.3. Phylogenetic Analyses and Investigation of Positive Selection Patterns

Multiple sequence alignments were performed with MAFFT [32], using the method FFT-NS-I in the online version (https://mafft.cbrc.jp/alignment/server/index.html). Phylogenetic trees for complete genomes or partial sections of homologous genomic regions were inferred with MrBayes v3.2.6 [33], modeling the distribution of mutations by general time-reversible evolutionary model, allowing for heterogeneity among sites distributed under a gamma distribution, with four categories and an extra category for invariable sites (GTR+Γ+I). Other priors were used with MrBayes defaults. The lengths of the chains were set to 2 × 10^6^ states, to assure convergence of the variability between runs to <0.05; also, after executions the effective sample sizes (ESS) of all model parameters were examined to assure ESS >100. Species delineation between clades of sequences were identified by following the ICTV criteria [3].

Additionally, we examined positive selection across all FAdV species, except for FAdV-B due to the low number (*n* = 2) of representative genomes. A ratio of nonsynonymous-to-synonymous nucleotide substitutions (ω = dN/dS) >1 was considered to identify sites under positive selection, using MrBayes, with a chain length of 5 × 10^5^ states to assure the ESS >100 for all parameters. Based on a sliding window analysis with 32 and 16 codons as window and step size, respectively, significance was assigned to the number of positively selected sites per window, using a binomial distribution assessing the probability of a site to be under positive selection (ratio of the total number of sites under positive selection to the total number of sites in the genome). Furthermore, we performed a gene-by-gene analysis of the number of sites (ω > 1) as a function of total codons in each annotated ORF across the genomes of all investigated species.

### 2.4. Genomic and Antigenic Composition of FAdV-D and FAdV-E Strains

The genomic similarities among new and available sequences of FAdV-E and FAdV-D strains were analyzed with SimPlot v3.5.1 [34], using prototype sequences of each extant serotype (CR119, YR36, TR59, and 764, as well as 685, SR49, A-2A, and 380; see Supplementary Table S1) as reference.

Based on our finding of FAdV-E as the most diversified of all investigated species, we used it as a platform to explore genomic variability and consequences on antigenic composition in more detail.

Informative regions were inferred from a sliding window analysis on the alignment of all FAdV-E genomes (using a 1400 nt window size, determined on the basis of a median pairwise distance >0.001 in 100% of the windows). Similarity among regions was estimated by comparing the topological distance between the respective phylogenetic trees, using Robinson–Foulds [35]. Then, the topological distance was used to distinguish between regions that provided virtually similar information about the genome and others that were more informative, thus allowing to select groups of phylogenetically nonredundant regions that could distinguish among different strains.

Additionally, in order to parse sites dictating antigenic variation, all currently sequenced FAdV-E strains were examined with the method of proteotyping adapted from Obenauer et al. [36]. Similar to previous studies in HAdVs, we used this approach to visualize each individual strain’s amino acid signatures across the major antigenic determinants, penton base, hexon, and fiber. Amino acid signatures were derived from clade-guided sequence alignments, indicating sites with a polymorphism relative to the most frequently occurring residue in that position with a frequency-based color code.

### 2.5. Coevolution and Recombination Analyses

Multiple genome alignments were analyzed with the suite of recombination detection algorithms in RDP v4.58 [37], for evidence of recombination events between the available FAdV-D and FAdV-E genomes, respectively. The considered methods were RDP [38], GENECONV [39], Chimaera [40], MaxChi [41], Bootscan [42], SiScan [43], and 3Seq [44], with a *p* threshold <0.01 and requiring the signal to be detectable simultaneously by at least three methods, as suggested in other studies, to reduce the false positive rate [40,45]. The effects of recombination events in the phylogeny of sections of the genome were assessed with a compatibility matrix similar to that described previously by Heath [46], where the compatibility of two windows with a 500 nt size and 50 nt per step is defined as the normalized Robinson–Foulds distance [47] between the corresponding neighbor-joining phylogenetic trees under a Tamura–Nei evolutionary model; thus, the compatibility reflects the similarity between the phylogenies for any two genome windows ranging from 0 (identical) to 1 (completely dissimilar). We also included the analysis of recombination breakpoints performed by RDP4, with a sliding window of 1000 nt with a one-nucleotide-step and 1000 permutations for estimating the statistical support of the breakpoint distribution.

### 2.6. Cross-Neutralization Testing

Cross-neutralization assays using type-specific antisera against an extensive panel of prototypes from FAdV-D and FAdV-E were performed to validate sequencing results. Tested field isolates additionally included three FAdV-E strains with novel, uniquely composed genomes, and one FAdV-D from a set of field strains with high sequence identity. Monospecific FAdV antisera were generated in specific pathogen-free chickens (Lohmann Tierzucht GmbH, Cuxhaven, Germany) immunized intramuscularly with a mixture of inactivated (1% formaldehyde) virus and adjuvant (GERBU LQ no. 3000, GERBU Biotechnik GmbH, Heidelberg, Germany). The procedures on experimental birds were discussed and approved by the institutional ethics committee and the national authority, according to §26 of the Law for Animal Experiments, Tierversuchsgesetz 2012–TVG 2012 (license numbers GZ 68.205/0158-WF/V/3b/2014, GZ 68.205/0044-WF/V/3b/2016).

Antibody titers were predetermined in a microtiter assay on CEL cells, testing all samples in duplicates. Heat inactivated, serially diluted (1:8–1:16,384) sera were incubated with 100 TCID_50_ of virus. After five days at 37 °C in 5% CO_2_, the wells were investigated for cytopathic effect. For cross-neutralization testing, sera with a standardized concentration of 20 serum units, and consecutive doubling dilutions were analyzed under the test conditions outlined above.

An 8-fold difference in titer compared to the homologous value served as the threshold for serotype differentiation [48,49]. Reciprocal relationships between certain strains of interest were further quantified by the Archetti and Horsfall’s formula [50].

## 3. Results

### 3.1. Molecular Phylogeny and Positive Selection Patterns of FAdVs

The next generation sequencing efforts rendered 27 new FAdV genome sequences with ca. 170× read depth median and the read length averaging 300 bp for all samples.

**Figure 1:**
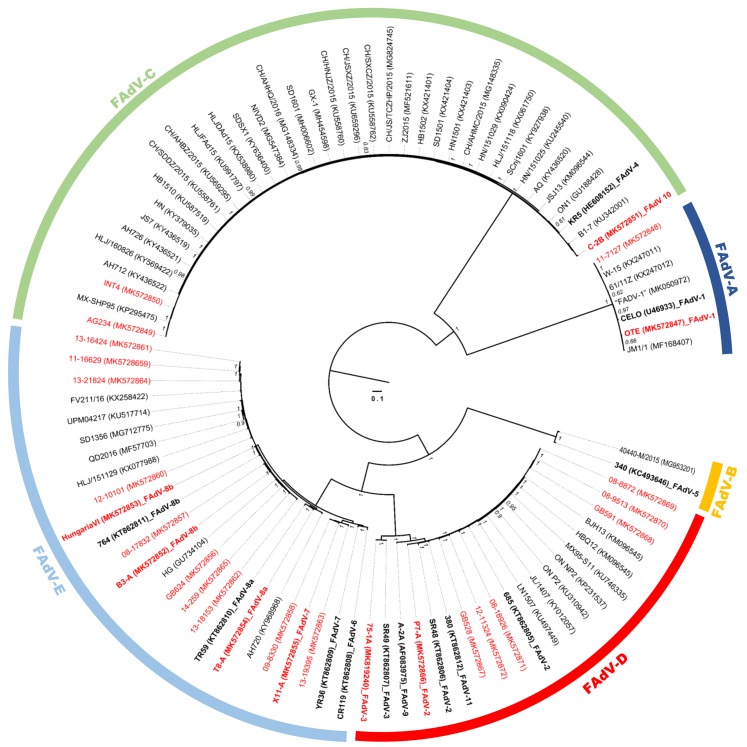
Phylogenetic tree of all currently available FAdV whole-genome sequences inferred with Bayesian methods. Strains sequenced in this study are indicated in red; all other sequences were retrieved from GenBank. Reference strains are shown in bold, with the official type affiliation included after the strain identifier. Branch lengths correspond to the ratio of nucleotide substitutions per site (see the scale). Posterior probability is indicated for values greater than 0.6, and the tree was rooted at the midpoint.

The phylogenetic tree on the basis of all available FAdV genomes showed a division into major groups, corresponding to the five known species and confirming the newly sequenced strains as members of FAdV-D or FAdV-E (Figure 1). Other sequenced genomes, extending our analyses, belonged to FAdV-A (reference strain OTE and field strain 11-7127) or FAdV-C (C-2B, prototype of the hitherto incomplete FAdV-10, and the virulent/*in vitro*-attenuated FAdV-4 pair AG234/INT4 [51]).

Newly sequenced FAdV-D field isolates were highly similar (≥97.4%) to reference genomes of both FAdV-2 and -11, conforming also to other recently published FAdV-D genomes. Of the remaining two types, represented only by historical reference strains, FAdV-3 (strains SR49, 75-1A) showed the greatest divergence (around 10%) to all other genomes, while FAdV-9 (A-2A) diverged by less than 5% from its most distant relatives in the FAdV-2/-11 cluster.

Contemporary FAdV-E field strains were most closely related (≥98% identity) to the official type members of FAdV-8b (764, B3-A and HungariaVI), or -8a (TR59 and T8-A). Only field strains 09-8330 and 13-19395 formed a separate monophyletic group together with newly sequenced prototype X11-A, with FAdV-6 (CR119) and -7 (YR36) reference genomes as their closest neighbors (≥95.5%).

Furthermore, the last so-far incompletely sequenced type, FAdV-10 (strain C-2B), was confirmed to have high similarity (>98%) across its entire genome to official FAdV-4 members. Noteworthily, this finding also applied to a so-far unsequenced segment of C2-B from 52/55K to hypothetical 9.1K, which includes the gene for the serologically relevant penton base.

**Figure 2:**
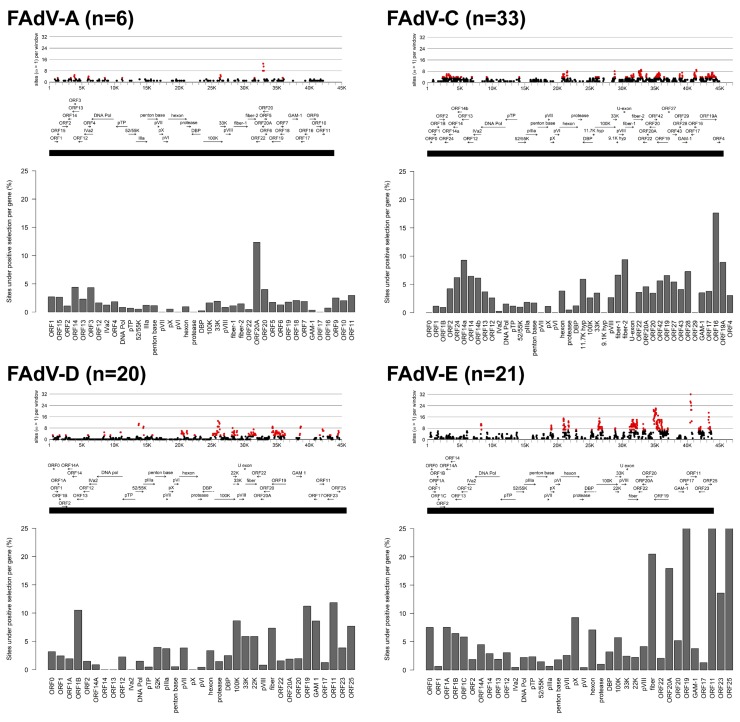
Comparison of mutational signatures of all FAdV species included in the analysis (except FAdV-B). The upper panels show the distribution of positive selection across the genome, marked in 1 kbp intervals along the x-axis. Dots indicate the number of sites under positive selection (dN:dS ratio >1) per window (window size = 32 codons), with significantly high counts (*p* < 0.01) represented by red dots. The bar charts below indicate the percentage of sites under positive selection for each annotated gene, with gene order and position labeled along the black horizontal line.

Analysis of the distribution of positively selected sites (ω > 1) across FAdV genomes exposed differences in the mutational landscapes of distinct species (Figure 2). With a 1.4% genome average of positively selected sites, FAdV-A was the most conserved species. Windows with peak counts for sites under positive selection were identified in ORF20A, whose content of positively selected codons relative to total gene length was higher than in any other annotated gene of FAdV-A (12.4% versus ≤4.4%).

In FAdV-C, featuring a genome-wide positive selection rate of 3.7%, windows indicating a high site-specific density of codons under positive selection mapped mainly to the terminal regions (ORF14a at the left genome end, and ORFs 20A, 19, 28, 16, and 19A at the left genome end), the area of the hexon loops-1 and -2, the 33K gene, and to both fiber genes. Of note, positively selected codons in the FAdV-C genome having experienced mutations in the newly sequenced strain pair AG234/INT4 were attributed to only four genes, ORF24, fiber-2, ORF19, and ORF16, indicating these as possible targets of virulence-modulatory selection pressures.

FAdV-E had the highest genome-wide positive selection rate (6.7%), compared to 3.8% in FAdV-D, which represents the most similar species counterpart in terms of gene contents and number of total codons in the genome considered for the analysis. Nevertheless, sites with significant counts of positive selection followed a characteristic distribution pattern in both species; the most prominent peaks included ORFs 19, 11, and 25 at the right genome end, fiber, the 5′-end of 100K, and a double peak indicating hexon loops-1 and -2. Additionally, common sites for positive selection in FAdV-D and FAdV-E were the overlapping encoding regions for 22K and 33K, and the 3′-end of the DNA polymerase gene. Further sites in which species-specific positive selection occurred were the genes for GAM-1, 52K, and pIIIa, and several left-terminal ORFs (in FAdV-D), as well as ORF20A and the pX and DBP genes (in FAdV-E).

In a gene-by-gene comparison, ORF19, ORF11, ORF25, and fiber also corresponded to the highest-scoring candidates for percentage-positive selection in both species. Other genes identified in this analysis, which did not necessarily concur with the narrow peaks of site-specific positive selection in some genes, were ORF1B, 100K, and GAM-1 (FAdV-D), as well as ORF20A and ORF23 (FAdV-E).

### 3.2. Genomic and Antigenic Composition of FAdV-D and FAdV-E Strains

The newly sequenced genomes of FAdV-D and FAdV-E ranged in size between 43,026 and 43,550 and between 43,560 and 44,342 bp, respectively. All of the strains featured the characteristic genome layout with 37 ORFs [8], which are identical between FAdV-D and FAdV-E, but distinct to the ORF contents of the remaining species.

**Figure 3:**
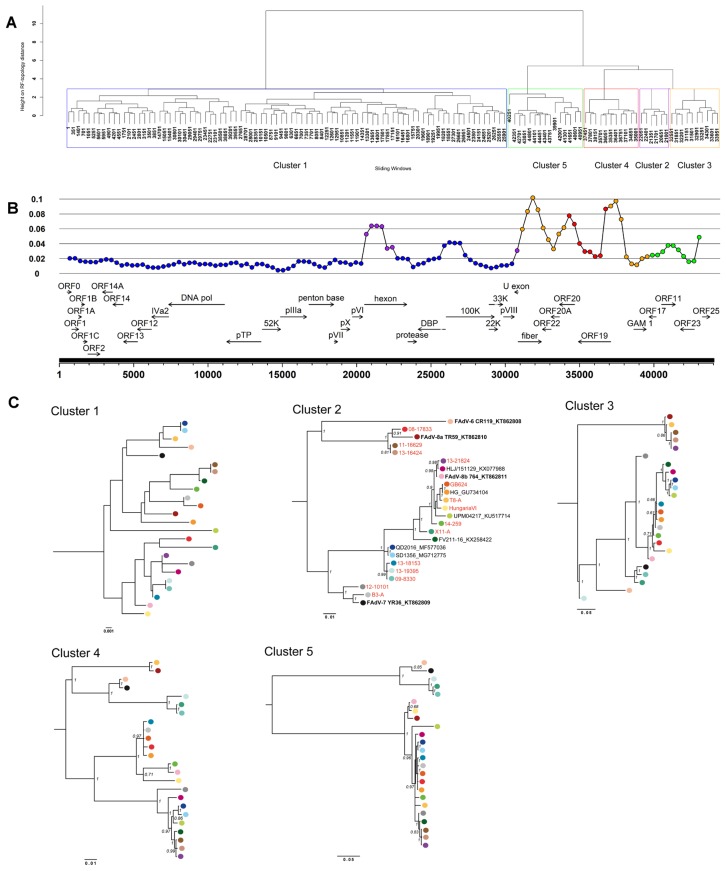
Analysis of informative sites in the FAdV-E genome, based on a sliding window analysis (window size = 1400 nt) on the alignment of all available complete sequences from this species. (**A**) Clustering of analyzed windows (indicated by the first nucleotide position of each window in the alignment) according to their phylogenetic content. Individual clusters, defined by windows with similar phylogenetic contents, are boxed in distinct colors and numbered as indicated at the bottom. (**B**) The regions contained in each cluster, plotted to a genome-wide map of diversity. Cluster-affiliation is indicated by the color-code assigned in (**A**). (**C**) Comparison of the topologies for each informative region. Individual colors mark the position of each strain across all topologies, revealing discordant phylogenetic grouping. Bootstrap support is shown for values >0.6. The scale bars below indicate the ratio of nucleotide substitutions per site for each phylogenetic tree.

Despite high global conservation between strain members of either species (≥90.0% nt identity), divergence of up to 27% occurred in four defined genomic segments with noniterative phylogenetic contents, determined on the basis of the most divergent species, FAdV-E. Two of these segments centered on antigenic domains, one comprising hexon, and another one encompassing fiber and its adjacent ORFs 22, 20A, as well as the 3′-end of ORF20. The further two segments were located in the right terminus, one spanning ORF19 and the 5′-end of ORF20, and the other one a concatenate of ORFs 11, 23 and 25 (Figure 3A,B).

**Figure 4:**
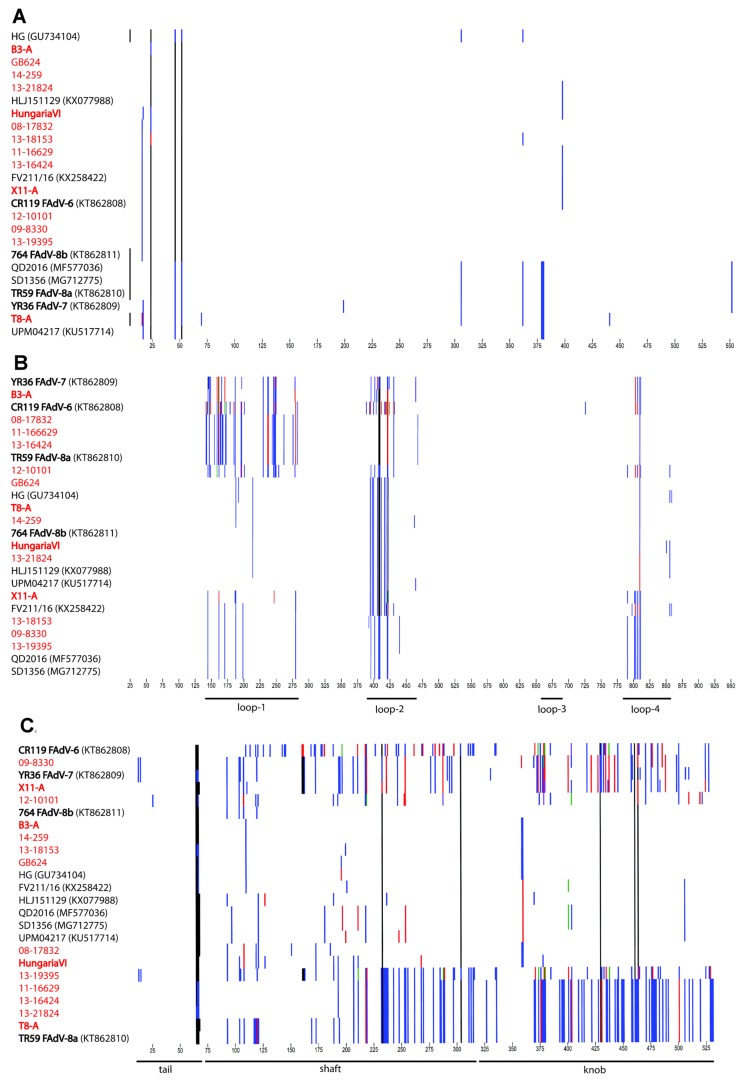
Proteotyping for the major antigenic determinants (**A**) penton base, (**B**) hexon, and (**C**) fiber, shown for all fully sequenced FAdV-E strains. An individual amino acid signature is curated for each of the strains indicated at the left margin, by marking residues that differ from the residue with the highest frequency in each position of the alignment with a colored vertical line. Blue indicates the second-most frequent type of residue, followed by red and green. Residues identical between all strains are represented in white, and gaps are in black. Relevant gene-specific landmarks are noted in their relative positions at the bottom of the graph.

Fine-scale analysis of the major antigenic domains showed that (i) polymorphisms in hexon mapped almost exclusively to the relative positions of loops-1, -2, and -4, as defined by Sheppard et al. [52], (ii) the fiber was entirely hypervariable, and (iii) penton base had a comparatively low degree of variation (<2.5% of the total protein), with serologically distinct strains being indistinguishable based on their amino acid sequence (Figure 4A–C).

**Figure 5:**
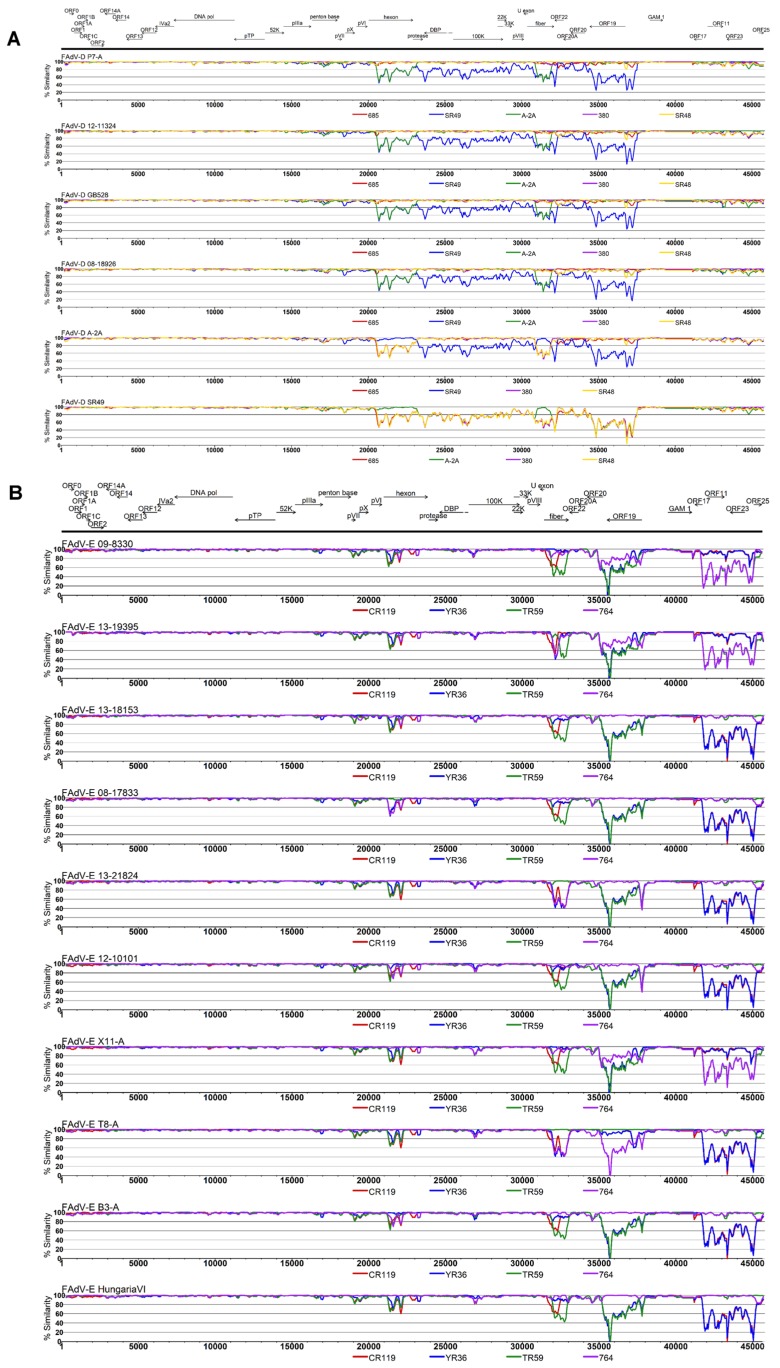
SimPlots for genomes of (**A**) all newly sequenced FAdV-D strains, as well as putative recombinant reference strain A-2A, and (**B**) all newly sequenced FAdV-E strains described in this study. Each panel shows the genome-wide similarity between the analyzed genome (indicated in the header of the panel) and selected reference sequences (bottom of the panel). Nucleotide positions in the genome are marked on the horizontal axis, and the genome annotation is provided on top of the panels for each species. Similarities were calculated with the Timura–Nei 93 (TN93) pairwise distance algorithm in a sliding window size of 1000 nt and a step size of 500 nt.

Compared to already published reference genomes, at least four FAdV-E field isolates, as well as three of the four newly obtained FAdV-E prototype strains had unique compositions, evident from incongruent phylogenetic relationships between informative genome regions (Figure 3C) and crossovers in their SimPlot profiles (Figure 5).

Strain 09-8330 was the only contemporary isolate exhibiting a close relationship to FAdV-7 in the fiber (98.7% identity), while its hexon was equidistantly related to reference strains from both FAdV-8b and -7 (97% and 96.7%, respectively). Its highest-scoring hexon relatives 13-18153 and 13-19395 (99.9–100% identity) had a similar intermediate relationship with both -8b and -7. Furthermore, 09-8330 and 13-19395 also shared unique phylogenetic relationships in ORF19, resembling only newly sequenced reference strain X11-A. In a right-terminal segment, which encompassed ORFs 11, 23, and 25, these three strains again diverged notably from a cluster containing all remaining field strain genomes, with closest relationship only to prototypes YR36 and CR119.

Additionally, strain 13-19395 showed greatest divergence from all other analyzed FAdV-E genomes, with a unique sequence pattern in its fiber (≤89.3% identity to all recognized types). Based on proteoprofiles, an unprecedented fiber composition, with highest similarity to FAdV-7 throughout the first half of the fiber, followed by a switch to -8a like motifs in the shaft domain, and a knob shared only with FAdV-6, which was encountered in 13-19395.

Strains 08-17832 and 13-21824 had typical FAdV-8a or -8b hexons, respectively, paired with a fiber of the opposite specificity, while their right genome terminus clustered consistently with the majority of other field strains (with the aforementioned exceptions, 09-8330 and 13-19395). Likewise, the newly sequenced prototype T8-A possessed mixed 8b- and 8a-specific hexon and fiber genes. However, its ORF19-segment revealed a unique clustering with FAdV-8a reference strain TR59, distinguishing these two genomes from all others.

Mixed hexon and fiber specificities were also found in prototype strain B3-A, with a type -7 like hexon and an -8b like fiber. Only reference strain HungariaVI consistently grouped with FAdV-8b in both domains. The fourth reference genome provided by our study, X11-A, was related to FAdV-7 only in its fiber gene (98.3%). Although its hexon sequence shared high overall percentage identities with FAdV-8b (≥98.0%), its proteoprofile distinguished by singular motifs in loop-1 and -2, and a loop-4 pattern which was only present in three other strains of our study (09-8330, 13-18153 and 13-19395).

Similarly, 12-10101, the only IBH field isolate with highest nt identity to FAdV-7, contained several mutations, mainly within the loop-1, compared to its closest relative. Besides this, its fiber, despite having greatest similarity to FAdV-8b (96.6%), contained the second most polymorphisms relative to any other investigated fiber (after FAdV-6 type strain CR119). Based on its right-terminal genome portion, 12-10101 clustered together with the majority of other investigated FAdV-E field strains.

Compared to FAdV-E, less crossovers were encountered in the genomic profiles of FAdV-D strains. The newly sequenced official FAdV-2 prototype P7-A had high identities to reference genomes from FAdV-2 and -11 (685, 380, SR48), conforming with the close relationships among those two types (97.8–98.1% nt identity). All presently available genomes from FAdV-D field strains also grouped in the FAdV-2/-11 cluster, with 97.4% identity between the most distant isolates.

With 99.7% identity between newly sequenced reference strain 75-1A and the only other available FAdV-3 representative SR49, FAdV-3 showed high intratype conservation, and, at the same time, the greatest divergence from all other FAdV-D members. The most noticeable common feature of the FAdV-3 strains was their unique ORF19, sharing only around 65% aa identity with all remaining FAdV-D counterparts. Another 12 kB segment, which was uniquely diversified in FAdV-3 vs. all other strains, spanned from hexon to the end of fiber open-reading frames. Two particular stretches of this segment, mapping to the entire hexon and fiber shaft, shared highest identity with FAdV-9 type representative A-2A, which was otherwise closely related to the FAdV-2/-11 strains.

### 3.3. Coevolution and Recombination Analyses of FAdV-D and FAdV-E

**Figure 6:**
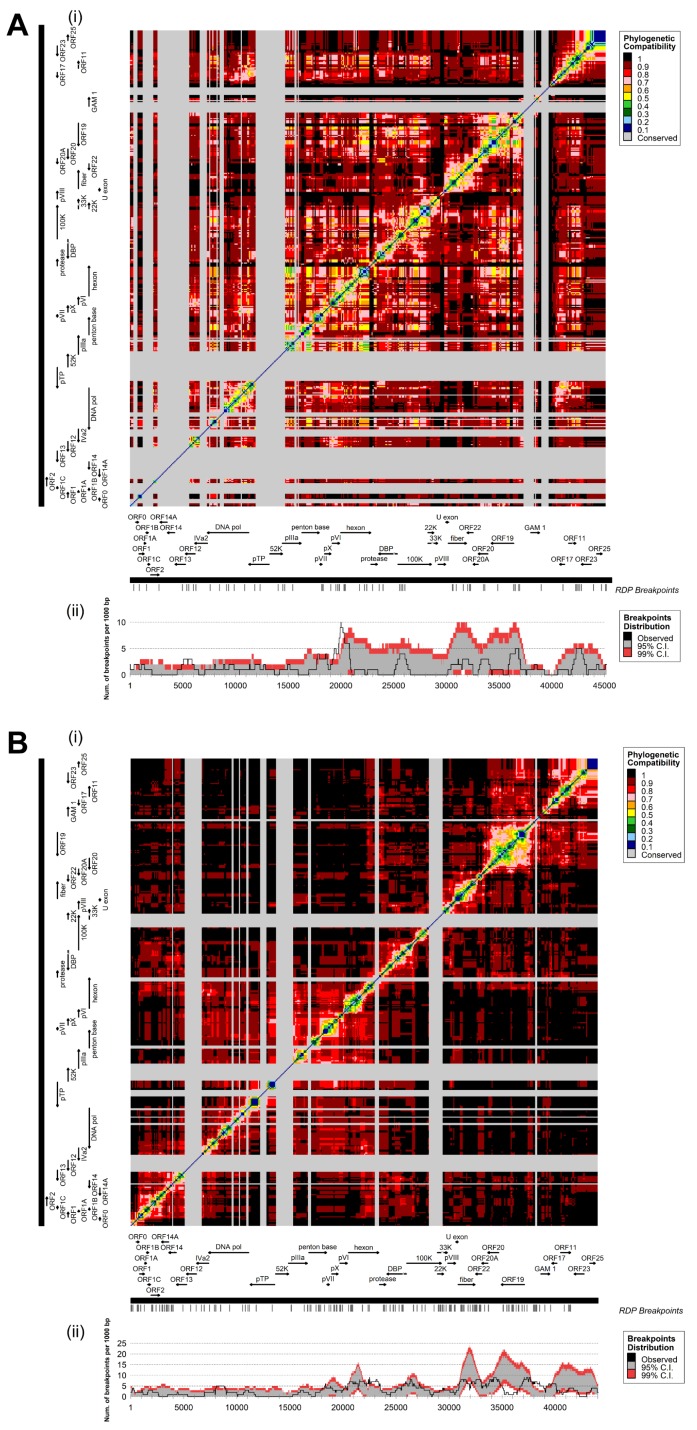
Coevolutionary and recombination analysis for (**A**) FAdV-D and (**B**) FAdV-E. Panel (i) shows the degree of coevolution at the intersection of any two regions of the genome (plotted along the horizontal and the vertical axes), inferred from similarity of the phylogenetic information for each window against the phylogenetic information in a different window (window size = 1000 nt, step size = 100 nt). With increasing similarity, the index between two windows decreases. Gray areas indicate windows for which the phylogenetic content was insufficiently diversified for comparison (Robinson–Foulds symmetric distance <0.1). Panel (ii) shows the genome-wide distribution of recombination breakpoints. Annotations of the analyzed species are provided in the genomic maps at the upper margin of the graph. All detectable breakpoint positions are indicated by small vertical lines at the top. The number of breakpoints (solid line) was calculated by moving 1000 nt windows in 1 nt steps along the alignment. Recombination hotspots were predicted where the number of observed breakpoints exceeds the local 99% confidence interval (CI), represented by the red area.

Coevolutionary matrices of FAdV-D and FAdV-E were characterized by block-like modules along the plot diagonal, conforming to the positions of the most variable genes, whose internal evolutionary concertation was contrasted by the low phylogenetic signal (with limited inference of coevolutionary relationships) in surrounding regions of the genome (Figure 6A,B, panel i). In both species, the most strongly coevolved areas encompassed ORFs 25, 19, and 11, as well as hexon and fiber. At the same time, these internally correlated genes exhibited a high degree of phylogenetic disconnection between them. Furthermore, boundaries of several of the identified blocks co-localized with breakpoints determined by recombination analysis.

Altogether, 47 and 117 recombination events were detected in FAdV-D and FAdV-E, respectively. Even though breakpoints were distributed throughout the genome, recombination focused in major hotspot locations, defined by boundaries with the 99% CI for local breakpoint estimates expected to occur randomly. In FAdV-D, a sharp, prominent signal centered on position 20,009, which maps directly upstream of the hexon gene (Figure 6A, panel ii). Furthermore, the whole surrounding region, including the pVI gene and the 5′-terminus of hexon, contained a recombination hotspot. Additional peaks were located in the genomic area of the left-terminal ORFs 12 and 13, and at the penton base 3′-terminus.

Likewise, in FAdV-E, hexon constituted a major hotspot for recombination, extending to the adjacent intergenic regions and genes (Figure 6B, panel ii). Additional breakpoint accumulations above the 95% CI occurred in FAdV-E at both sides of the fiber gene. Another putative hotspot included GAM-1 and its preceding intergenic region that contains a 33-bp iteration of varying copy numbers (tandem repeat-1). Periodically repeated sequence motifs were also observed in the vicinity of predicted hotspots in the DNA polymerase, the overlapping coding region for 100K, 22K and 33K, as well as the inverted terminal repeats (ITRs), indicating a possible association between repetitive genomic elements and recombination.

### 3.4. Cross-Neutralization Assays

**Figure 7:**
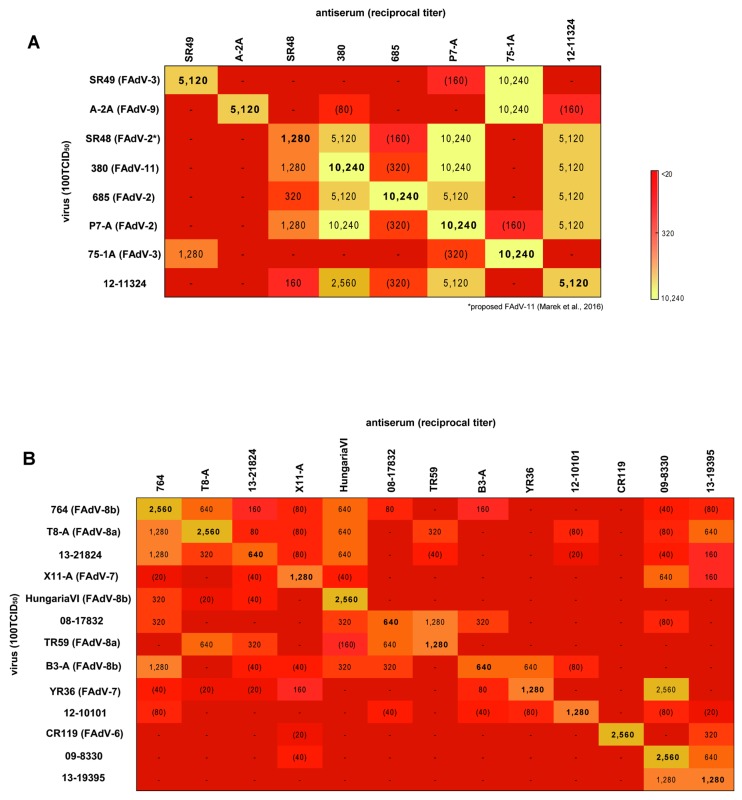
Cross-neutralization between reference strains (official serotype designation is given in parentheses) and selected field strains of (**A**) FAdV-D and (**B**) FAdV-E. Heatmaps show the extent of neutralization (represented as the mean reciprocal titers from duplicate testing) of antisera standardized to 20 serum units against 100 TCID_50_ of virus. The horizontal bar shows the color key. Boldface values along the diagonal represent the homologous titers; reactions judged to be minor (below an 8-fold homologous:heterologous ratio) are indicated in parentheses.

Of all available FAdV-D reference strains, only SR49 and A-2A exhibited reactions within their corresponding serotype, namely FAdV-3 and FAdV-9 (Figure 7A). Serum against reference strain 75-1A, officially designated as FAdV-3, neutralized both SR49 (FAdV-3) and A-2A (FAdV-9) to a similarly high degree, but mutual recognition occurred only with SR49. Frequent cross-reactivities were recorded between FAdV-2 and FAdV-11, where mutual neutralization occurred between all relevant prototypes. The only exception from this was reference strain 685, which, despite being recognized by all other FAdV-2/-11 antisera, showed only strict homologous reactivity.

Within FAdV-E, no serological relationship was observed between the prototypical representatives CR119 (FAdV-6), YR36 (FAdV-7), TR59 (FAdV-8a), and 764 (FAdV-8b) (Figure 7B), confirming their separate serological identity (henceforth termed as “genuine” type strains). On the contrary, cross-reactivities of two of these genuine type representatives, T8-A (reacting with FAdV-8a and -8b) and B3-A (reacting with FAdV-7 and -8b) occurred, based on bidirectional neutralization in each case. Although antiserum against strain HungariaVI elicited neutralizing activity against all tested viruses with 8b-specific hexon and/or fiber genes, HungariaVI was recognized in turn only by antiserum against strain 764. Besides this, some cross-reactivity was also noted against genuine FAdV-8a strain TR59, but the titer was below the 8-fold homologous:heterologous threshold.

Of all tested prototypes, X11-A was the least broadly reactive, with only a weak one-sided reaction against genuine FAdV-7 YR36.

Consistent with their mixed antigenic composition determined by sequence analysis, two of the tested FAdV-E field strains cross-reacted with FAdV-8a and -8b reference strains. However, dominant serological relationships were either with FAdV-8a (08-17832 and prototype TR59 neutralized each other to the same titer as their homologous virus, compared to an interrelatedness of only 12.5% with prototype 764, according to the Archetti–Horsfall index), or FAdV-8b (reciprocal neutralization between 13-21824 and 764, as opposed to a one-sided reaction with TR59). Furthermore, the two field strains were serologically distinct from each other, and in their reactivities with the newly sequenced prototypes (08-17832 showed mutual reactivity with B3-A, and 13-21824 with T8-A).

Field strains 09-8330 and 13-19395 were 50% interrelated based on Archetti-Horsfall and were not recognized by any of the heterologous test sera. However, while serum against 13-19395 neutralized CR119 (FAdV-6), as well as the antigenically mixed strains T8-A and 13-21824, serum against 09-8330 recognized YR36 (FAdV-7) in a strong, yet unilateral reaction. Furthermore, these two strains were the only ones to recognize X11-A, which shared certain motifs in the hexon with both, as well as fiber specificity with 09-8330. The last field strain under study, 12-10101, showed no heterologous reaction above the 8-fold threshold to any other tested strain, indicating its position as a possibly new and self-standing serotype.

## 4. Discussion

Natural recombination, and its primacy for adenovirus evolution, is recognized mainly in mastadenoviruses, with extensive documentation in certain HAdV species [53,54,55,56,57,58,59]. Occasional discoveries are also reported across other species of the genus, e.g., porcine, canine, and bat AdV [60,61,62]. These include examples of allegedly less common interspecies and intergenus recombination events, which were also described for a novel, non-chicken AdV mosaïc of sequences with different aviadenovirus origin [63]. Although this finding is indicative to a widespread exchange between genomes of aviadenoviral species, our current study is the first one to report recombination in FAdVs, with consequences on biological properties, specifically cross-neutralization.

The identified recombination events were confined to the intraspecies level, occurring in the type-rich FAdV species D and E; moreover, a preliminary recombination analysis reported negative results on potential interspecies events. With at least nine variant genomes in a total of 13 investigated strains, FAdV-E contained an exceptionally high proportion of recombinants. This suggests that the propensity for (detectable) recombination events increases with the number of genetically diverse types present in a species, while, concurrently, recombination itself is a driving force to create mosaicism between types. It is noteworthy that FAdV-A and FAdV-C were excluded from the recombination analysis of this study due to the relatively high conservation among available strains and the consequent limited interpretation of detected recombination events. Furthermore, FAdV-A and FAdV-C showed an average 0.006 pairwise evolutionary distance in both species; on the other hand, FAdV-D and FAdV-E showed 0.03 and 0.04 average pairwise evolutionary distances, respectively. Differences in the recombinogenic potential of FAdVs might be dictated by their unique genomic landscape, which differs not only from adenoviruses of other genera, but also among members of FAdV, depending on the species they belong to.

A major hallmark of FAdVs are double fibers of the viral capsid, transcribed in a species-specific manner from either one (FAdV-B, FAdV-D, and FAdV-E), or two separate genes (FAdV-A and FAdV-C) [13,31,64,65], while the remaining central part of the genome is largely conserved in relation to the mastadenovirus E2 and late (L1–L5) regions. Since the other mastadenovirus transcript families E1A/E1B, E3, and E4, which encode important immune modulatory functions, lack homologues in FAdVs, similar effectors are presumed among their poorly characterized terminal contents [66]. This notion is supported by species-specific variations in terminal ORFs, which emphasize the distinctiveness of FAdV-A and FAdV-C from the FAdV-D/FAdV-E complex, conforming with major differences in the biology of these species and the induced diseases [7].

Extending the earlier observation that phylogenetically differentiated sites are sharply concentrated within the FAdV genome [8], we further sorted these sites into four unique, non-redundantly informative complexes, consisting of hexon, fiber, and two right-terminal concatenates. Particularly in FAdV-E genomes, the effect of internally correlated elements that evolve autonomously could be visualized by a modular segmentation of the interregional coevolutionary map. Furthermore, coevolutionary patterns reflected an overall independence between both genome termini and the mid-genomic section, which is further split into at least two parts, segregating hexon from fiber. Several of the identified segments were accompanied by recombination hotspots at their boundaries, while they also conformed with genes, or gene regions, prone to positive selection.

Collectively, these findings indicate a modular exchange between FAdV genomes, analogous to the mechanism described earlier in HAdVs [58], in any of the regions subjected to adaptive pressures; unsurprisingly, these include the antigenically exposed hexon and fiber, but also a subset of terminal ORFs specific for FAdVs at the rightmost genome end (ORF19, which is shared between all species, and ORFs 11 and 25, which are common only to FAdV-D and FAdV-E). Recombination signals were also noticed in regions which are not known to be translated, among them the right-terminal ORF20/20A family. Interestingly, this alludes to findings in a different species, FAdV-A, where we detected an exceptionally divergent, 3′-truncated ORF20A, present only in reference strain CELO (currently annotated ORF21 with an erroneous sequence; data not shown). Considering that ORF20A could act as a regulatory element of downstream mRNA [67], and since it was the only noteworthy genomic variation between apathogenic CELO and other FAdV-A strains, this transcription unit merits further attention, also in the context of recombination.

Compared to FAdV-E, recombination in FAdV-D may still be underestimated due to limited genome data from other types than the IBH-causing, and thus most rewarded, FAdV-2 and -11. Additionally, the relatedness between FAdV-2 and -11 is as close as between members of their own type, questioning a sequence-based demarcation between them. In FAdV-D, we propose FAdV-9 prototype A-2A as a recombinant, with FAdV-2/-11 related sequences surrounding two segments acquired from FAdV-3. One of those segments comprised the fiber shaft-encoding region, ca. 250–1310 nt downstream of the poly-G stretch. The latter position marked a switch at the predicted shaft–knob boundary, a breakpoint occasionally also reported in HAdVs [68]. Although we showed hypervariability throughout all parts of the FAdV fiber, its complex conformation remains obviously intact during such a recombination event, indicating the least disruptive effect for the protein with breakpoints at an interdomain junction. The most notable divergence between FAdV-3 and other type members existed in ORF19. Similar to a recent finding in FAdV-B [69], this gene (and its predicted protein) was as divergent within the species as from its closest relative of another species, possibly suggesting that it had been derived in independent events from a reservoir outside the species.

Preferential occurrence of recombination between types of one species, but not beyond the intraspecies level, may be explained with constraints due to species-specific genome contents. Since coinfection of the same cell is necessary for recombination, such constraints likely include differences in the fiber, a molecule that relegates the virus to a specific infectious pathway or target tissue. From a clinical perspective, this prioritizes recombination within the IBH complex and urges awareness for mixed-strain infections, increasing the risk of recombination if they involve FAdV-E or FAdV-D.

Recombinant FAdV-E strains addressed in this study were isolated from IBH outbreaks, or induced disease in experimental settings [70], underlining their clinical relevance. Antigenic segments with parental origin of types -6 and -7, which are usually not linked to IBH, were also found among field isolates (12-10101, 09-8330, and 13-19395), although experimental data on the pathogenicity of the latter two strains are unavailable so far. Nevertheless, this exposes a possible gap in the recognition of “types” and their association with disease.

Mixed genetic identities in hexon and fiber concurred with intermediate serological reactivities of such strains, reflecting that both hexon and fiber participate in FAdV neutralization. Provided that the reaction partners were not sufficiently related in one antigenic domain, this could be compensated for by the other; however, when the reaction partners shared only their fiber specificity, recognition tended to be slightly weaker. This suggests that fiber ranks second to hexon in neutralization, at least in the response against nonreplicating virus as in our setting, possibly explained by the numerical overrepresentation of hexon in the capsid. Likewise, prototypes T8-A, B3-A and X11-A, confirmed by sequencing as recombinants, were serological intermediates between two serotypes, warranting their cautious use for reference purposes. Our own results for the historical prototypes were aligned with observations by the original researchers [71,72], with a few exceptions. Results for X11-A (ATCC VR-835) were different from those obtained earlier with the similarly denominated X11. In this case, ours and the historical setting seem to contain two strains with separate genetic identities [5], which are both serologically affiliated to FAdV-7 as a consequence of antigenic reshuffling. Strain HungariaVI was less broadly reactive in our hands than previously reported, with no reaction partners outside FAdV-8b, which could be reconciled with its genomic composition. The observation that HungariaVI is poorly neutralized by homologous antisera, thus defining a prime strain, resembled findings in earlier studies. However, the molecular basis of this remains unclear, highlighting that distance-based criteria alone are not always fully conclusive for serological reactivities. For instance, despite having less than 3.4% divergence from its closest relative in both antigenic domains, 12-10101 defined an entirely new type as per cross-neutralization. Its proteoprofiles suggested that, in addition to recombination, this strain had enough variation in areas putatively responsible for eliciting neutralizing antibodies to separate it sufficiently from any other type. Proteoprofiles also exposed a novel fiber composition in a single field strain that reacted with FAdV-6, with which it shared similarities only in the knob but not in other domains. As another intra-fiber recombinant, besides the one identified in FAdV-D, this strain confirmed that specificity of the fiber C-terminus alone can influence the response pattern.

Its strict self-neutralization, despite being recognized by all FAdV-2/-11 members, could highlight FAdV-2 prototype strain 685 as an antigenic variant with prime relationships to FAdV-2/-11. Reported cross-reactivity between the FAdV-3 and -9 prototypes 75-1A and A-2A [71] was limited in our study to a strong, but unilateral activity of 75-1A against A-2A. Since both hexon and penton base aa sequences of 75-1A were highly similar to strain SR49, which did not neutralize A2-A, there remained only few possible residues in fiber, specifically its knob domain, which could be responsible for this difference in neutralization. In the context of other reference strains, which had not been available for joint testing in previous studies, categorization of FAdV-2 (defined by 685) and FAdV-9 (defined by A-2A) as self-standing types illustrates a certain conundrum brought about by using viruses with partial (prime) relationships or recombinants for type determination.

Cross-neutralization results also strengthen the proposed reclassification of prototype SR48 into FAdV-11 [8], and the merging of FAdV-2 and -11 into a single type, supporting data published by Steer et al. [73]. Likewise, the sequence-based similarity of FAdV-4 and FAdV-10 argues for FAdV-C as a quasi-monotype species. This is, according to our findings, only sufficiently contested by a phylogenetic stand-alone position of FAdV-10 strain C-2B in the hexon gene, but not in other serological determinants, possibly explaining cross-reactivities with FAdV-4 [73,74].

Along with these controversies on the actual number of constitutive types, the extent of recombination in other species than FAdV-E remains somewhat debatable, further strengthening the outstanding role of recombination in FAdV-E.

Based on the enrichment of positively selected genes, other than those involved in antigenic variation, in recombination, we hypothesize a strategy for evading cellular components of host defense, analogous to recombination events described in the E3 region of HAdVs [57,75]. The evolutionary concertation of ORFs 11, 23, and 25 possibly argues for a synergistic activity, although none of the putative products have been characterized so far. Interestingly, clustering of strains by their clinical background was observed for ORF25, with all isolates from IBH outbreaks separated against isolates with atypical (non-IBH related) or no clinical findings reported, indicating a possible constraint on virus biology by this gene. Homologies of ORF11 to the CD4 precursor of T-cells, and ORF19 to a virulence factor of Marek’s disease virus [75], have provided intriguing hypotheses on their function; however, it is noted for ORF19 that positive selection is confined to its distal part, suggesting a functional domain outside the (proximally encoded) lipase equivalent.

Based on the extension of our findings to serotyped but hitherto unsequenced reference strains, and a recently deposited genome of an FAdV-E field strain (KY968968) in the public database, we propose that FAdV recombination is spatially and temporally widespread. While this explains the difficulties to taxonomically assign several official prototypes [2], it also raises concerns for current typing practices. Some of the sites which we showed in our study to capture global diversity of FAdVs (e.g., ORF25) may be better associated with pathogenicity and tropism than hexon, justifying their incorporation into more complete identification strategies, with possible approaches toward a combined numerical system proposed for the classification of viruses [76].

Furthermore, knowledge about the existence of recombinants is crucial for vaccination studies, emphasizing the choice of vaccine and challenge strains as a subject of scrutiny. Additionally, possible recombination between targets of the immune system warrants a cautious interpretation of cross-protection results in already-existing studies.

## Supplementary Materials

The following is available online at https://www.mdpi.com/1999-4915/11/12/1094/s1. Table S1: FAdV strains investigated within this study.
