# Supplementary material for: Fowl Adenovirus (FAdV) Recombination with Intertypic Crossovers in Genomes of FAdV-D and FAdV-E, Displaying Hybrid Serological Phenotypes
Source: Viruses. 2019 Nov 26;11(12):1094. doi: 10.3390/v11121094 (PMC6950264; doi:10.3390/v11121094)
Supplement: Supplementary file 1 [file viruses-11-01094-s001.pdf]

Supplementary table 1. FAdV strains investigated within this study.

Strain name	GenBank accession no.	Serological relationship			Genome features		Strain source (country/year[b])	Obtained from[c]	Original reference
		Official reference designation	This study	Related strains in other studies[a]	Length (bp)	GC (%)			
**Newly sequenced strains**									
OTE	MK572847	FAdV-1	FAdV-1	CELO	43,816	54.2	Japan	Vaxxinova GmbH	[15]
11-7127	MK572848	n.a.[d]	FAdV-1	n.a.	43,795	54.3	Germany/2011	n.a.	[16]
AG234 (formerly IV4)	MK572849	n.a.	FAdV-4	n.a.	45,505	54.7	Mexico/1995	Dr. H.M. Hafez, Berlin	[17]
INT4[e]	MK572850	n.a.	n.d.[f]	n.a.	45,629	54.6	n.a.	n.a.	[18]
C-2B (VR-834)	MK572851	FAdV-10	FAdV-4/-10	KR5	45,554	54.6	USA	Dr. H.M. Hafez, Berlin	[19]
B3-A (VR-832)	MK572852	FAdV-8b	FAdV-7/-8b	764	44,161	58.0	USA	Vaxxinova GmbH	[19]
HungariaVI	MK572853	FAdV-8b	FAdV-8b/-8a	HungariaVI<br>764<br>TR59	44,037	57.8	Hungary	Dr. H.M. Hafez, Berlin	[20]
T8-A (VR-830)	MK572854	FAdV-8a	FAdV-8a/-8b	YR36 (one-way)<br>TR59<br>HungariaVI<br>764	44,047	57.9	USA	ATCC	[19]
X11-A (VR-835)	MK572855	FAdV-7	FAdV-7	YR36	43,947	57.8	USA	Vaxxinova GmbH	[19]
GB624	MK572856	n.a.	n.d.	n.a.	44,017	57.9	USA/1998	Dr. H.M. Hafez, Berlin	[21]
08-17832	MK572857	n.a.	FAdV-8a/-8b	n.a.	43,807	57.9	France/2008	n.a.	[2]
09-8330	MK572858	n.a.	FAdV-7	n.a.	43,450	57.7	Hungary/2009	n.a.	[2]
11-16629	MK572859	n.a.	n.d.	n.a.	43,845	57.8	Spain/2011	n.a.	[14]
12-10101	MK572860	n.a.	new serotype	n.a.	44,112	57.9	Germany/2012	n.a.	[14]
13-16424	MK572861	n.a.	n.d.	n.a.	42,198	57.8	France/2013	n.a.	[14]
13-18153	MK572862	n.a.	n.d.	n.a.	44,342	58.0	Greece/2013	n.a.	[14]
13-19395	MK572863	n.a.	FAdV-6	n.a.	42,476	57.8	Germany/2013	n.a.	[14]
13-21824	MK572864	n.a.	FAdV-8b/-8a	n.a.	43,600	57.8	France/2013	n.a.	[14]
14-259	MK572865	n.a.	n.d.	n.a.	43,760	57.8	France/2014	n.a.	[14]
P7-A (VR-827)	MK572866	FAdV-2	FAdV-2/-11	-	43,056	53.2	USA	Vaxxinova GmbH	[19]
75-1A (VR-831)	MK819240	FAdV-3	FAdV-3/-9	SR49	43,550	52.9	N. Ireland	Vaxxinova GmbH	[22]
GB528	MK572867	n.a.	n.d.	n.a.	43,462	53.2	Switzerland/1998	Dr. H.M. Hafez, Berlin	n.a.
GB591	MK572868	n.a.	n.d.	n.a.	43,322	53.1	Germany/1998	Dr. H.M. Hafez, Berlin	n.a.
08-8872	MK572869	n.a.	n.d.	n.a.	43,541	53.3	Germany/2008	n.a.	[2]
08-9513	MK572870	n.a.	n.d.	n.a.	43,026	53.2	Germany/2008	n.a.	[2]
08-18926	MK572871	n.a.	n.d.	n.a.	43,310	53.2	Austria/2008	n.a.	[2]
12-11324	MK572872	n.a.	n.d.	n.a.	43,405	53.2	Austria/2012	n.a.	[14]
**Already available sequences**									
CELO (Phelps)	U46933<br>MK572875*	FAdV-1	FAdV-1	OTE	43,804	54.3	USA/1957	Dr. H.M. Hafez, Berlin	[23]
W-15	KX247011	n.a.	n.a.	n.a.	43,849	54.3	Poland	n.a.	[24]
61/11z	KX247012	n.a.	n.a.	n.a.	43,854	54.3	Poland/2011	n.a.	[24]
JM1/1	MF168407	FAdV-1	n.a.	n.a.	43,809	54.3	Japan/2000	n.a.	[25]
“FAdV-1”	MK050972	n.a.	n.a.	n.a.	43,739	54.2	China	n.a.	n.a.

340	KC493646	FAdV-5	FAdV-5	TR-22	45,781	56.5	N. Ireland	Dr. H.M. Hafez, Berlin	[22]
40440-M/2015 Debrecen	MG953201	n.a.	n.a.	n.a.	45,743	56.4	Hungary	n.a.	[26]
KR5	HE608152	FAdV-4	FAdV-4/10	C-2B	45,810	54.6	Japan	Dr. H.M. Hafez, Berlin	[15]
B1-7	KU342001	n.a.	n.a.	n.a.	45,622	54.6	India/2011	n.a.	n.a.
ON1	GU188428	FAdV-4**	n.a.	n.a.	45,667	54.6	Canada/2004	n.a.	[27]
JSJ13	KM096544	n.a.	n.a.	n.a.	43,755	54.8	China/2013	n.a.	[10]
AQ	KY436520	n.a.	n.a.	n.a.	43,723	54.8	China/2016	n.a.	n.a.
HLJ/151118	KX061750	n.a.	n.a.	n.a.	43,612	54.8	China/2015	n.a.	n.a.
HN/151025	KU245540	n.a.	n.a.	n.a.	43,613	54.8	China/2015	n.a.	n.a.
HN/151029	KX090424	n.a.	n.a.	n.a.	43,614	54.8	China/2015	n.a.	n.a.
SCnj1601	KY927938	n.a.	n.a.	n.a.	43,719	54.8	China/2016	n.a.	[28]
ZJ2015	MF521611	n.a.	n.a.	n.a.	43,717	54.8	China/2015	n.a.	n.a.
NIVD2	MG547384	n.a.	n.a.	n.a.	43,719	54.8	China	n.a.	n.a.
HN1501	KX421403	n.a.	n.a.	n.a.	43,662	54.8	China/2015	n.a.	n.a.
SD1501	KX421404	n.a.	n.a.	n.a.	43,634	54.9	China/2015	n.a.	n.a.
HB1502	KX421401	n.a.	n.a.	n.a.	43,621	54.8	China/2015	n.a.	n.a.
SDSX1	KY636400	n.a.	n.a.	n.a.	43,630	54.8	China/2015	n.a.	n.a.
HLJDAd15	KX538980	n.a.	n.a.	n.a.	43,722	54.8	China/2015	n.a.	n.a.
HLJFAd15	KU991797	n.a.	n.a.	n.a.	43,720	54.8	China/2015	n.a.	n.a.
CH/AHMC/2015	MG148335	n.a.	n.a.	n.a.	43,721	54.8	China/2015	n.a.	n.a.
CH/JS/TCZHP/2015	MG824745	n.a.	n.a.	n.a.	43,721	54.8	China/2015	n.a.	n.a.
HB1510	KU587519	n.a.	n.a.	n.a.	43,721	54.8	China/2015	n.a.	[11]
HN	KY379035	n.a.	n.a.	n.a.	43,724	54.8	China	n.a.	n.a.
JS7	KY436519	n.a.	n.a.	n.a.	43,723	54.8	China/2015	n.a.	n.a.
AH712	KY436522	n.a.	n.a.	n.a.	43,725	54.8	China/2016	n.a.	n.a.
AH726	KY436521	n.a.	n.a.	n.a.	43,723	54.8	China/2016	n.a.	n.a.
HLJ/160826	KY569422	n.a.	n.a.	n.a.	43,723	54.8	China/2016	n.a.	n.a.
CH/AHHQ/2016	MG148334	n.a.	n.a.	n.a.	43,721	54.8	China/2016	n.a.	n.a.
GX-1	MH454598	n.a.	n.a.	n.a.	43,721	54.8	China/2017	n.a.	n.a.
SD1601/FAdV-4	MH006602	n.a.	n.a.	n.a.	43,723	54.8	China/2016	n.a.	n.a.
CH/AHBZ/2015	KU569295	n.a.	n.a.	n.a.	43,721	54.8	China/2015	n.a.	[12]
CH/JSXZ/2015	KU569296	n.a.	n.a.	n.a.	43,723	54.8	China/2015	n.a.	[12]
CH/HNJZ/2015	KU558760	n.a.	n.a.	n.a.	43,725	54.8	China/2015	n.a.	[12]
CH/SXCZ/2015	KU558762	n.a.	n.a.	n.a.	43,721	54.8	China/2015	n.a.	[12]
CH/SDDZ/2015	KU558761	n.a.	n.a.	n.a.	43,725	54.8	China/2015	n.a.	[12]
MX-SHP95	KP295475	n.a.	n.a.	n.a.	45,641	54.7	Mexico/1995	n.a.	[29]
685	KT862805<br>MK572874*	FAdV-2	FAdV-2/-11	SR48<br>380	43,430	53.2	N. Ireland	Dr. H.M. Hafez, Berlin	[22]
380	KT862812<br>MK572873*	FAdV-11	FAdV-2/-11	685<br>SR48	43,302	53.2	N. Ireland/1971	Dr. H.M. Hafez, Berlin	[22]
SR48	KT862806	proposed<br>FAdV-11[g]	FAdV-2/-11	685<br>380	43,632	53.3	Japan	Dr. H.M. Hafez, Berlin	[15]
SR49	KT862807	FAdV-3	FAdV-3	A-2A<br>75-1A	43,337	52.7	Japan	Dr. H.M. Hafez, Berlin	[15]
A-2A	AF083975	FAdV-9	FAdV-9	SR49	45,063	53.7	USA/1961	Dr. H.M. Hafez, Berlin	[19]
BJH13	KM096546	n.a.	n.a.	n.a.	43,961	53.5	China/2013	n.a.	[10]
HBQ12	KM096545	n.a.	n.a.	n.a.	44,080	53.5	China/2012	n.a.	[10]

MX95-S11	KU746335	n.a.	n.a.	n.a.	44,326	53.69	Mexico/1995	n.a.	[30]
ON NP2	KP231537	n.a.	n.a.	n.a.	45,193	54.07	Canada/2005	n.a.	[31]
ON P2	KU310942	n.a.	n.a.	n.a.	44,377	53.72	Canada/2005	n.a.	[31]
JL/1407	KY012057	n.a.	n.a.	n.a.	44,054	53.53	China/2014	n.a.	n.a.
LN/1507	KU497449	n.a.	n.a.	n.a.	44,111	53.58	China/2015	n.a.	n.a.
CR119	KT862808	FAdV-6	FAdV-6	-	43,810	57.90	Japan	n.a.	[15]
YR36	KT862809	FAdV-7	FAdV-7	X11-A	43,525	57.78	Japan	n.a.	[15]
TR59	KT862810	FAdV-8a	FAdV-8a	58	43,287	57.94	Japan	n.a.	[15]
764	KT862811	FAdV-8b	FAdV-8b	T8-A	43,666	57.81	N. Ireland	n.a.	[22]
HG	GU734104	FAdV-8b**	n.a.	n.a.	44,055	57.92	Canada	n.a.	[32]
AH720	KY968968	n.a.	n.a.	n.a.	39,057	58.06	China/2016	n.a.	n.a.
FV211-16	KX258422	n.a.	n.a.	n.a.	43,976	57.92	Peru/2016	n.a.	[33]
HLJ/151129	KX077988	n.a.	n.a.	n.a.	43,534	57.76	China/2015	n.a.	n.a.
QD2016	MF57703	n.a.	n.a.	n.a.	43,632	57.87	China/2016	n.a.	n.a.
SD1356	MG712775	n.a.	n.a.	n.a.	44,454	58.14	China/2016	n.a.	n.a.
UPM04217	KU517714	n.a.	n.a.	n.a.	44,072	57.93	Malaysia/2004	n.a.	[34]

[a]Indicated for previously documented reactions exceeding the 8-fold threshold for homologous:heterologous titer difference (settings may vary between individual studies).
[b]Year of isolation is given if documented.
[c]Indicated for virus strains obtained from external sources.
[d]n.a., not applicable.
[e]*In vitro*-attenuated daughter strain of AG234.
[f]n.d., not done.
[g]According to Marek et al., 2016.
*GenBank accession numbers for full genomic sequences that have been corrected in this study.
**Reference strains based on sequence identities instead of cross-neutralization.
